# Volumetric Change in the Masseter and Lateral Pterygoid after Mandibular Setback

**DOI:** 10.3390/jpm12050820

**Published:** 2022-05-18

**Authors:** Jae Hyun Kang, Dong Sun Shin, See Woon Kim, Hun Jun Lim, Bong Chul Kim

**Affiliations:** Department of Oral and Maxillofacial Surgery, Daejeon Dental Hospital, Wonkwang University College of Dentistry, Daejeon 35233, Korea; wogus31001@gmail.com (J.H.K.); sdssoft@gmail.com (D.S.S.); tologst@naver.com (S.W.K.); hun216@wku.ac.kr (H.J.L.)

**Keywords:** prognathism, masseter muscle, pterygoid muscle, orthognathic surgery, CT X ray

## Abstract

In this study, we evaluated changes in the masseter and lateral pterygoid muscles in the prognathic mandible group after a mandibular setback by comparing the volume-to-length ratios. Preoperative and postoperative 1-year computed tomography was used to calculate the volume-to-length ratio of the lateral pterygoid and masseter muscle in 60 Korean individuals. Three-dimensional images were reconstructed, the results of which showed no significant differences in the volume-to-length ratios of the masseter and lateral pterygoid muscles after a mandibular setback (*p* > 0.05). This result was found for both vertical ramus osteotomy and sagittal split ramus osteotomy, and for both males and females. No significant differences in the volume-to-length ratio of the masseter and lateral pterygoid muscles were found up to 1 year after a mandibular setback. Therefore, this study can contribute to the prediction of soft-tissue profiles after mandibular setback.

## 1. Introduction

Mandibular prognathism is a morphological abnormality occurring in the oral and maxillofacial regions, resulting in aesthetic and functional problems [1,2,3,4]. Although corrective and surgical methods are available for the treatment of mandibular prognathism, surgery is the most appropriate treatment for most adults [5,6,7]. However, young patients can be treated with orthodontic methods, which include timing treatment options, growth prediction of the mandible, and control of the growth patterns. Therefore, many studies have been conducted on elucidating the growth pattern of the mandible and the factors affecting it [1,2,3,8].

In our previous study [1], the mandibles of class I (normal mandible) and class III (prognathic mandible) patients were divided into five skeletal units (coronoid, condyle, angle, symphysis, and body), and each skeletal unit was compared between the class I and class III groups. The study results showed that the body and condyle units of the class III group were thinner and longer than those of the class I group. When dependent on sex, the differences were significant in the condyle unit for the male group and in the body unit for the female group.

In our previous studies [2,3], we compared the masseter and lateral pterygoid muscles of class I and class III patients. The results of the studies showed that the volume-to-length ratios of the masseter and lateral pterygoid muscles in the class I group were larger than those in the class III group. In addition, the superficial and deep heads of the masseter in the class I group had a more vertical tendency than those of the class III group, and the lateral pterygoid muscle of the class I group had a more horizontal tendency than that of the class III group.

Based on these previous studies, we hypothesized that the volume-to-length ratios of the masseter and the lateral pterygoid muscles may increase when the mandibular protrusion was surgically corrected. The aim of this research is to determine how the masseter and lateral pterygoid muscles of the class III group change after a mandibular setback through orthognathic surgery. Therefore, in this study, the volume-to-length ratios of the masseter and lateral pterygoid muscles in patients with mandibular prognathism before and after a mandibular setback were compared.

## 2. Materials and Methods

### 2.1. Participants

Sixty Korean participants (30 male and 30 female) who visited Wonkwang University Daejeon Dental Hospital were evaluated. Computerized tomography (CT) images were obtained for patients wishing to undergo orthognathic surgery. Dental and skeletal measurements were conducted with three-dimensional (3D) CT scans. The inclusion criteria included the following: age between 18 and 29 years, willingness to undergo 3D CT of the skull, and presence of full dentition excluding the permanent third molars. The exclusion criteria were congenital deformities, chronic periodontitis, and a history of jaw trauma. CT images were obtained before the operation and one year after. The jaws of 60 participants were analyzed. This study was approved by the institutional review board of Daejeon Dental Hospital, Wonkwang University (W2202/003-002).

### 2.2. Classification

Preoperative Delaire [9] and Steiner analyses were conducted to classify the skeletal patterns of the patients, and only mandibular prognathism was included in this study. For the Delaire analysis, we evaluated the angle between the C1 plane and Me-based F1 plane. The Me-based F1 plane spans the area from the naso-fronto-maxillary point to the menton point, and the C1 plane is the horizontal reference plane. The Steiner analysis was conducted to evaluate the sella-nasion-A point angle (SNA), sella-nasion-B point angle (SNB), and A point-nasion-B point angle (ANB). These values were calculated on the midsagittal plane [10] of the 3D CT images. Based on high C1-Me-based F1 angles and high SNB values, the participants were classified as having a prognathic mandible (83.59° ± 1.07° for SNB and 92.63° ± 1.07° for C1-Me-based F1 on average (mean ± standard deviation)).

A mandibular setback was conducted on all participants with either vertical ramus osteotomy (VRO) or sagittal split ramus osteotomy (SSRO). After surgery, the patients were allowed to function immediately or undergo a short period of intermaxillary fixation.

First, we compared the changes in volume-to-length ratios after a mandibular setback, regardless of the surgical method used. Second, we compared changes in volume-to-length ratios after a mandibular setback according to the surgical method applied (VRO and SSRO). Third, we compared the changes in volume-to-length ratios after a mandibular setback according to sex.

### 2.3. CT Imaging and 3D Image Reconstruction

A three-dimensional (3D) analysis was conducted using 3D CT to evaluate the masseter and lateral pterygoid muscles. The CT images were recorded at the Wonkwang University Daejeon Dental Hospital with a SOMATOM Definition Dual Source CT (Siemens, Forchhelm, Germany) under the following imaging conditions: a visual field of view of 20.8 cm, a voltage of 100 kV, an imaging speed of 76 mAs, a scanning time of 1 s, and a thickness of 0.5 mm. The participants were instructed to remain motionless and maintain a centric occlusion to avoid changes in masseter and lateral pterygoid muscle volumes. The CT cross-sectional images of all patients were saved in the Digital Imaging Communication in Medicine format, and the 3D images were reconstructed.

**Figure 1:**
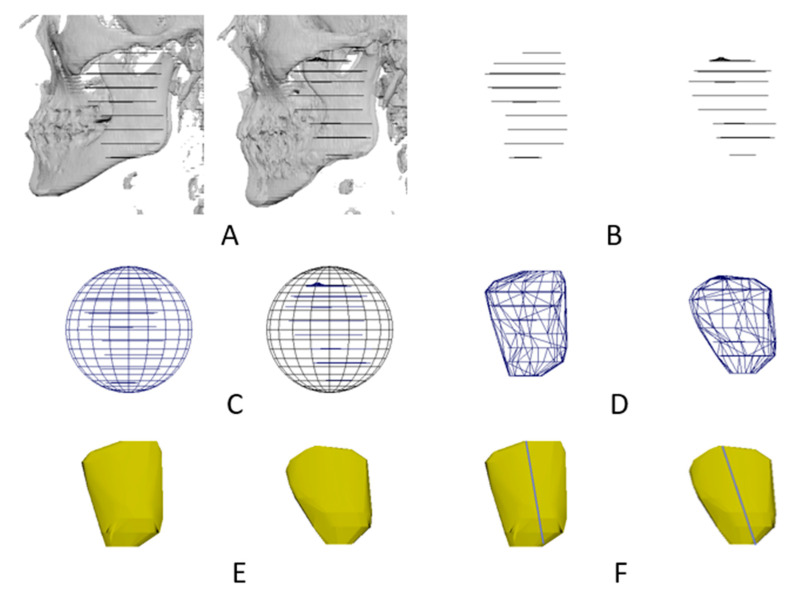
Surface reconstruction of a masseter muscle before (**left**) and after (**right**) a mandibular setback. The masseter muscle and skull were initially surface reconstructed. (**A**) Transparent skull revealing the initial surface models of the masseter muscle. (**B**) Initial surface models of the masseter muscle. (**C**) A polyhedron generated and (**D**) wrapped around the initial surface models of the masseter muscle. (**E**) Realistically and smoothly reconstructed surface model of the masseter muscle. (**F**) Length of the masseter muscle measured using a ruler.

Mimics software was used for a surface reconstruction of the masseter and lateral pterygoid muscles. There were options for low-, middle-, and high-quality surface reconstructions. Mid- and high-quality shapes, by contrast, were difficult to process in the subsequent step, owing to the large file size. As a result, the only option was to use a low-quality solution. However, because whole segmentation was not applied, the surface models of the masseter and lateral pterygoid muscles were not smoothly or realistically reconstructed. Outlines were sparsely drawn. Therefore, the masseter and lateral pterygoid muscles were segregated and not joined in the surface models. An additional processing was conducted to make the surface models of the masseter and lateral pterygoid muscles realistic and smooth. A polyhedron was created and wrapped in the initial surface models of the masseter and lateral pterygoid muscles; this procedure was performed with Maya version 2022 (Autodesk Inc., San Rafael, CA, USA). Thus, the surface models of the masseter and lateral pterygoid muscles were accurately reproduced (Figure 1).

### 2.4. Volume and Length Calculation

The volume of the masseter and lateral pterygoid muscles was measured with the “Poly Volume” tool in the Maya bonus tools and the “Head-up Display” tool in the Maya standard display menu. The lengths of the masseter and lateral pterygoid muscles were measured with the surface models of the ruler. In the “Transform Attributes” window, the ruler length was presented and the value was acquired. To reduce the time required, Python scripts created in Maya were used to perform these repetitive tasks.

Because the masseter and lateral pterygoid muscle shapes exhibit individual differences, a characteristic ratio was calculated by dividing the volume by the length. The calculations were conducted with Microsoft Excel.

### 2.5. Method Error

The method error when selecting the reference points on the 3D CT images (measured as intraobserver and interobserver errors) was evaluated by two of the authors, who randomly selected 30 reference points. At 3-week intervals, 30 reference points were selected on the 3D CT images. The results were assessed with Dahlberg’s formula and analyzed statistically with the intraclass correlation (ICC) as follows:

*E*_(*x* or *y*_, _or *z*)_ = √∑[(*x_n_* − *x_n_*_−1_)^2^ or (*y_n_* − *y_n_*_–1_)^2^ or (*z_n_* − *z_n_*_−1_)^2^]/2*N*,

where *x_n_* denotes the positional value of point *x_n_,* and *x_n_*_−1_ is the positional value of point *x_n_*_−1_ in the *x* coordinate.

### 2.6. Statistical Analysis

The mean ± standard deviation of the results was calculated. An independent *t*-test was conducted to determine the statistical significance. Statistical significance was set at *p* < 0.05. All statistical analyses were performed with IBM SPSS Statistics (version 23.0; IBM Corp., Armonk, NY, USA).

## 3. Results

### 3.1. Reliability of Measurements

The intraobserver error ranged between 0.2 and 0.7 mm for a reappearance of the reference points. The ICC with 95% confidence distances was 0.989 (*p* > 0.0001) for the intraobserver reliability and 0.931 (*p* > 0.0001) for the interobserver reliability.

### 3.2. Changes in Volume-to-Length Ratios after Mandibular Setback

There were no significant differences in the volume-to-length ratios after mandibular setback surgery (Table 1). There were no significant changes in the masseter ratio between the preoperative (352.2 ± 9.8) and postoperative (366.6 ± 9.9) groups, or in the lateral pterygoid ratio between the preoperative (291.1 ± 11.2) and postoperative (288.2 ± 10.9) groups. An independent *t*-test revealed no significant changes in the volume-to-length ratios after a mandibular setback (*p* > 0.05).

### 3.3. Changes in Volume-to-Length Ratios after Mandibular Setback According to Surgical Methods

There were no significant differences in the volume-to-length ratios after a mandibular setback according to the surgical method applied (Table 2). In the VRO group, there were no significant changes in the ratio of preoperative (369.7 ± 12.7) to postoperative (380.7 ± 12.8) masseter, or in the ratio of preoperative (295.4 ± 13.8) to postoperative (288.2 ± 15.7) lateral pterygoid. In the SSRO group, there were no significant changes in the ratio of preoperative (334.7 ± 14.3) to postoperative (352.4 ± 14.8) masseter, or in the ratio of the preoperative (286.8 ± 17.8) to postoperative (288.2 ± 15.5) lateral pterygoid. The independent *t*-test revealed no significant changes in the volume-to-length ratios after a mandibular setback according to the surgical method used (*p* > 0.05).

### 3.4. Changes in Volume-to-Length Ratios after Mandibular Setback According to Sex

There were no significant differences in the volume-to-length ratios after a mandibular setback according to sex (Table 3). In the male group, there were no significant changes in the ratio of preoperative (395.0 ± 9.4) to postoperative (392.2 ± 14.2) masseter, or in the ratio of preoperative (305.1 ± 15.1) to postoperative (310.7 ± 15.5) lateral pterygoid. In the female group, there were no significant changes in the ratio of preoperative (309.3 ± 13.1) to postoperative (341.0 ± 12.2) masseter, or in the ratio of preoperative (277.0 ± 16.3) to postoperative (265.6 ± 14.6) lateral pterygoid. An independent *t*-test revealed that there were no significant changes in the volume-to-length ratios after a mandibular setback according to sex (*p* > 0.05).

## 4. Discussion

In 1968, Moss first proposed the concept of the mandible as a functional unit composed of a dentoalveolus, coronoid, body, angle, condyle, and symphysis [11,12]. The author also suggested the concept of a functional matrix, i.e., the soft tissue surrounding the bone, and theorized that the bone growth is regulated by functional matrix [13]. Several studies have reported a relationship between orofacial morphology and masticatory muscles. Marques et al. reported that the brachycephalic pattern has a greater masticatory force and that the dolichocephalic pattern has a relatively weak masticatory force, through the calculation of the muscular work and mechanical advantage of the temporalis muscle [14]. In addition, Van Spronsen et al. suggested that the complex action of the mandibular muscles can induce a remodeling of the mandible. They found that the anterior vertical craniofacial dimensions were related to the orientation of the masticatory muscles [15]. Moreover, Sella-Tunis et al. showed that the shape of a wide mandible is related to the strength of the large masticatory muscles. They showed that larger muscles controlled for sizes correlated with a wider, more massive coronoid, more trapezoidal ramus, more curved basal arch, and a more rectangular body [16]. Finally, Gionhaku et al. and Kiliaridis described the relationship between brachycephalic appearance and a strong masticatory force. Gionhaku et al. reported that subjects with large medial pterygoid and masseter muscle volumes had small gonial angles, and flat mandibular and occlusal planes. Kiliaridis represented that an increase in the function of the masticatory muscles was related to the anterior-growth rotation pattern of the jaw [17,18].

Studies have also investigated the changes in the size and masticatory force of the masticatory muscles after orthognathic surgery. Katsumata et al. indicated that in mandibular prognathism, the cross-sectional area of the masses decreases after 3 months of mandibular setback but shows a tendency to return to normal after 1 year [19]. In addition, Ueki et al. reported that there are no significant differences in the cross-sectional area of the masseter in mandibular prognathism 1 year after SSRO in comparison with the preoperative area [20]. Trawitzki et al. also reported that when mandibular setback was conducted on patients with a class III dentofacial deformity, the thickness of the masseter muscle increased [21]. To date, most studies in this area have been conducted on the thickness and masticatory force of the masseter after a mandibular setback.

As such, most previous studies have concentrated on the masseter muscles. However, this study also investigated the changes in the lateral pterygoid muscle. As revealed in our previous studies [1,2,3], in mandibular prognathism, both the masseter muscle and the lateral pterygoid muscle showed significant differences from that of the normal mandible. To the best of our knowledge, no previous studies have examined the changes in the lateral pterygoid muscle after a mandibular setback. Moreover, a comprehensive comparison of surgical methods (VRO and SSRO) based on sex was also conducted in this study.

The results showed that the volume-to-length ratio of the masseter and lateral pterygoid muscles at 1 year after the mandibular setback did not show a significant difference compared with the preoperative value. This was the same regardless of whether VRO or SSRO was used as the surgical method. In addition, there were no significant changes between males and females. This result suggests that even if the mandibular protrusion was corrected through surgery, there was no significant change in the size of the masticatory muscle until 1 year after the operation. A limitation of this study is the insufficient follow-up period. We collected CT image data 1 year after mandibular setback. The results might differ in the future, and further studies are needed to confirm this hypothesis. Additionally, the change in muscle function after mandibular setback can be estimated by measuring the volume-to-length ratio of the masseter and lateral pterygoid muscles, but the changed function cannot be directly presented.

## 5. Conclusions

The results of this study showed that there were no significant differences in the volume-to-length ratio of the masseter and lateral pterygoid muscles until 1 year after mandibular setback.

## Figures and Tables

**Table 1:** Changes in volume-to-length ratios after mandibular setback.

	Preoperative (*n* = 60)	Postoperative (*n* = 60)	*p* Value
Ratio of masseter	352.2 ± 9.8	366.6 ± 9.9	0.301
Ratio of lateral pterygoid	291.1 ± 11.2	288.2 ± 10.9	0.853

Note: Values are presented as the mean ± SD, ratio = volume (mm^3^)/length (mm); abbreviation: SD, standard deviation, *p*: *p*-value of the paired *t*-test, * *p <* 0.05.

**Table 2:** Changes in volume-to-length ratios after a mandibular setback according to the surgical methods applied.

		Preoperative (*n* = 60)	Postoperative (*n* = 60)	*p* Value
VRO (*n* = 30)	Ratio of masseter	369.7 ± 12.7	380.7 ± 12.8	0.542
	Ratio of lateral pterygoid	295.4 ± 13.8	288.2 ± 15.7	0.732
SSRO (*n* = 30)	Ratio of masseter	334.7 ± 14.3	352.4 ± 14.8	0.392
	Ratio of lateral pterygoid	286.8 ± 17.8	288.2 ± 15.5	0.953

Note: Values are presented as the mean ± SD, ratio = volume (mm^3^)/length (mm); abbreviations: SD, standard deviation, *p*: *p*-value of the paired *t*-test, * *p <* 0.05, VRO: vertical ramus osteotomy, SSRO: sagittal split osteotomy.

**Table 3:** Changes in volume-to-length ratios after a mandibular setback according to sex.

		Preoperative (*n* = 60)	Postoperative (*n* = 60)	*p* Value
male (*n* = 30)	Ratio of masseter	395.0 ± 9.4	392.2 ± 14.2	0.869
	Ratio of lateral pterygoid	305.1 ± 15.1	310.7 ± 15.5	0.795
female (*n* = 30)	Ratio of masseter	309.3 ± 13.1	341.0 ± 12.2	0.083
	Ratio of lateral pterygoid	277.0 ± 16.3	265.6 ± 14.6	0.603

Note: Values are presented as the mean ± SD, ratio = volume (mm^3^)/length (mm); Abbreviations: SD, standard deviation, *p*: *p*-value of the paired *t*-test, * *p <* 0.05.

## Data Availability

The datasets generated and/or analyzed during the current study are available from the corresponding author upon reasonable request, subject to the permission of the institutional review boards of the participating institutions.

## References

[1] Mun S.H., Park M., Lee J., Lim H.J., Kim B.C. (2019). Volumetric characteristics of prognathic mandible revealed by skeletal unit analysis. Ann. Anat.-Anat. Anz..

[2] Yang J.H., Shin D.S., Yoo J.-H., Lim H.J., Lee J., Kim B.C. (2021). Anatomical characteristics of the masseter muscle in mandibular prognathism. Appl. Sci..

[3] Kim H., Shin D., Kang J., Kim S., Lim H., Lee J., Kim B. (2021). Anatomical characteristics of the lateral pterygoid muscle in mandibular prognathism. Appl. Sci..

[4] Badiali G., Lunari O., Bevini M., Bortolani B., Cercenelli L., Lorenzetti M., Marcelli E., Bianchi A., Marchetti C. (2021). Existence of a neutral-impact maxillo-mandibular displacement on upper airways morphology. J. Pers. Med..

[5] Park J.C., Lee J., Lim H.J., Kim B.C. (2018). Rotation tendency of the posteriorly displaced proximal segment after vertical ramus osteotomy. J. Cranio-Maxillo-Facial Surg..

[6] Alcañiz P., Pérez J., Gutiérrez A., Barreiro H., Villalobos Á., Miraut D., Illana C., Guiñales J., Otaduy M.A. (2021). Soft-tissue simulation for computational planning of orthognathic surgery. J. Pers. Med..

[7] Badiali G., Bevini M., Lunari O., Lovero E., Ruggiero F., Bolognesi F., Feraboli L., Bianchi A., Marchetti C. (2021). Psi-guided mandible-first orthognathic surgery: Maxillo-mandibular position accuracy and vertical dimension adjustability. J. Pers. Med..

[8] Kim K.J., Park J.H., Chang N.Y., Kim B.C., Chae J.M. (2021). Hemimandibular hyperplasia treatment with condylectomy and orthodontic camouflage treatment using miniplate. Am. J. Orthod. Dentofac. Orthop..

[9] Lee S.H., Kil T.J., Park K.R., Kim B.C., Kim J.G., Piao Z., Corre P. (2014). Three-dimensional architectural and structural analysis—A transition in concept and design from Delaire’s cephalometric analysis. Int. J. Oral Maxillofac. Surg..

[10] Kim H.-J., Kim B.C., Kim J.-G., Zhengguo P., Kang S.H., Lee S.-H. (2014). Construction and validation of the midsagittal reference plane based on the skull base symmetry for three-dimensional cephalometric craniofacial analysis. J. Craniofac. Surg..

[11] Moss M.L. (1968). A theoretical analysis of the functional matrix. Acta Biotheor..

[12] Enlow D.H., Moyers R.E. (1982). Handbook of Facial Growth.

[13] Moss M.L., Simon M.R. (1968). Growth of the human mandibular angular process: A functional cranial analysis. Am. J. Phys. Anthropol..

[14] Marques H.B., Richter F.F., Heck L., Xavier L.L., de Campos D. (2016). Biomechanical potential of the temporal muscle in brachyfacial and dolichofacial subjects: A study on dry mandibles. Orthod. Craniofac. Res..

[15] van Spronsen P.H., Koolstra J.H., van Ginkel F.C., Weijs W.A., Valk J., Prahl-Andersen B. (1997). Relationships between the orientation and moment arms of the human jaw muscles and normal craniofacial morphology. Eur. J. Orthod..

[16] Sella-Tunis T., Pokhojaev A., Sarig R., O’Higgins P., May H. (2018). Human mandibular shape is associated with masticatory muscle force. Sci. Rep..

[17] Gionhaku N., Lowe A. (1989). Relationship between jaw muscle volume and craniofacial form. J. Dent. Res..

[18] Kiliaridis S. (1995). Masticatory muscle influence on craniofacial growth. Acta Odontol. Scand..

[19] Katsumata A., Fujishita M., Ariji Y., Ariji E., Langlais R.P. (2004). 3d CT evaluation of masseter muscle morphology after setback osteotomy for mandibular prognathism. Oral Surg. Oral Med. Oral Pathol. Oral Radiol. Endod..

[20] Ueki K., Okabe K., Mukozawa A., Miyazaki M., Marukawa K., Hashiba Y., Nakagawa K., Yamamoto E. (2009). Assessment of ramus, condyle, masseter muscle, and occlusal force before and after sagittal split ramus osteotomy in patients with mandibular prognathism. Oral Surg. Oral Med. Oral Pathol. Oral Radiol. Endod..

[21] Trawitzki L.V., Dantas R.O., Elias-Júnior J., Mello-Filho F.V. (2011). Masseter muscle thickness three years after surgical correction of class III dentofacial deformity. Arch. Oral Biol..

